# Sleep home monitoring: a technical option!

**DOI:** 10.5935/1808-8694.20130049

**Published:** 2015-10-04

**Authors:** Edilson Zancanella

**Affiliations:** Coordinator of the Sleep Disorders Service - ORL/UNICAMP, Associate Editor - Bucco-Pharyngology and Sleep Medicine - BJORL.

Sleep Medicine is still a very young field in medicine, being recognized as a joint-working field, belonging to neurology, pneumology, psychiatry and otorhinolaryngology - according to a 2011-decision from the Federal Board of Medicine. The first certification in sleep medicine in Brazil happened in 2012, with 96 physicians certified, 50 of them being otorhinolaryngologists.

Brazilian otorhinolaryngologists have had a growing interest in this new field, and this has promoted the creation of an annual program in Sleep Medicine and Polysomnography by the ABORL - the only program available from these medical specialties - today in its 6^th^ edition. According to data from the 2012 ABORL Census, a large part of Brazilian ENTs manage patients complaining of snoring and sleep apnea, and these physicians are interested in updating their knowledge on this topic.

The prevalence of obstructive sleep apnea syndrome (OSAS) had its classic description in 1993^1^, affecting 4% of middle-aged men and 2% of middle-aged women. In a recent study carried out in 2010^2^ in São Paulo the authors report a mean value of 32.9% among men and women.

Data obtained from anthropometric measures and questionnaires may lead us to a diagnosis suggestive of OSAS; however, a definitive diagnosis and the severity assessment will only happen after sleep monitoring. The gold standard test to diagnose OSAS is the Type I polysomnography^3^ - an entire night test, carried out in a sleep laboratory under technical supervision. The large demand for this test and the lack of specialized facilities (sleep labs), even in large urban centers, make it difficult to diagnose the problem and, consequently, it delays treatment. All Brazilian labs have a long waiting list, both in private practice as well as in the SUS (Public Health Care system). The costs associated with setting up and maintaining a sleep lab are substantial, not only because of having to have a polysomnography technician, but also because of all the necessary setup. Another point that is always challenged by physicians and patients alike is if the results of the test really portray what happens when the patient is sleeping in his/her home.

Technological progress has brought about both a reduction in monitoring equipment dimensions, such as recording data from the entire night in the device itself, making them portable. However, there is a reduction in the number of physiological variables monitored by such equipment: options with oximetry data only - Type IV^3^; cardiorespiratory data - Type III^3^ - and even data similar to the gold standard - Type II^3^ - with all the items included in sleep architecture.

Sleep home monitoring (SHM) will become increasingly available and deserves to be detailed and analyzed from the viewpoint of practical applicability as well as that of the costs involved in its large scale use. The possibility of having SHM represents a real opportunity to broaden the availability of OSAS diagnosis and follow up, besides yielding a significant cost reduction - for it does not need to have the whole setup and the technician may play a less relevant role. Moreover, the patients submitted to home monitoring tend to prefer it over its lab counterpart.

A smaller number of channels available for home monitoring may not diagnose all types of sleep disorders; thus making it fundamental to have a full grasp of the possible differential diagnoses and the capabilities of the equipment to be employed. A proper training in sleep medicine enables the physician to know the types of monitoring devices available and for which type of problem they fit better. We recognize that the sleep lab plays its relevant role in the investigation of parasomnias, REM sleep behavioral disorders, PAP titration, and for those cases in which the predictive diagnosis is not confirmed. A fundamental issue to be stressed is that sleep monitoring, either in the lab or at home, still is a complementary test. Proper management requires clinical reasoning, a diagnostic hypothesis, and confirmation or not according to the test report. The quality of this report is something we must never take for granted!

Recurrent editorials in sleep medicine journals from the United States have highlighted the significant changes brought about by SHM popularization. Soon we will also have in Brazil most of the equipment used in SHM, and it will be up to us to have the knowledge and the technical capacity to indicate the best option for the patient under our management.
